# Persistent, and Asymptomatic Viral Infections and Whitefly-Transmitted Viruses Impacting Cantaloupe and Watermelon in Georgia, USA

**DOI:** 10.3390/v14061310

**Published:** 2022-06-15

**Authors:** Ismaila Adeyemi Adeleke, Saritha Raman Kavalappara, Cecilia McGregor, Rajagopalbabu Srinivasan, Sudeep Bag

**Affiliations:** 1 Department of Plant Pathology, University of Georgia, Tifton, GA 31793, USA; adelekeis@uga.edu; 2 Department of Horticulture, University of Georgia, Athens, GA 30602, USA; cmcgre1@uga.edu; 3 Department of Entomology, University of Georgia, Griffin, GA 30223, USA; babusri@uga.edu

**Keywords:** watermelon, cantaloupe, persistent virus, cucumis melo endornavirus (CmEV), cucumis melo amalgavirus (CmAV1), cucumis melo cryptic virus (CmCV), watermelon crinkle leaf-associated virus 1 (WCLaV-1), Georgia, USA

## Abstract

Cucurbits in Southeastern USA have experienced a drastic decline in production over the years due to the effect of economically important viruses, mainly those transmitted by the sweet potato whitefly (*Bemisia tabaci* Gennadius). In cucurbits, these viruses can be found as a single or mixed infection, thereby causing significant yield loss. During the spring of 2021, surveys were conducted to evaluate the incidence and distribution of viruses infecting cantaloupe (*n* = 80) and watermelon (*n* = 245) in Georgia. Symptomatic foliar tissues were collected from six counties and sRNA libraries were constructed from seven symptomatic samples. High throughput sequencing (HTS) analysis revealed the presence of three different new RNA viruses in Georgia: cucumis melo endornavirus (CmEV), cucumis melo amalgavirus (CmAV1), and cucumis melo cryptic virus (CmCV). Reverse transcription-polymerase chain reaction (RT-PCR) analysis revealed the presence of CmEV and CmAV1 in 25% and 43% of the total samples tested, respectively. CmCV was not detected using RT-PCR. Watermelon crinkle leaf-associated virus 1 (WCLaV-1), recently reported in GA, was detected in 28% of the samples tested. Furthermore, RT-PCR and PCR analysis of 43 symptomatic leaf tissues collected from the fall-grown watermelon in 2019 revealed the presence of cucurbit chlorotic yellows virus (CCYV), cucurbit yellow stunting disorder virus (CYSDV), and cucurbit leaf crumple virus (CuLCrV) at 73%, 2%, and 81%, respectively. This finding broadens our knowledge of the prevalence of viruses in melons in the fall and spring, as well as the geographical expansion of the WCLaV-1 in GA, USA.

## 1. Introduction

Cantaloupe and watermelon are economically important crops in Georgia; they are cultivated on an estimated average of 2400 acres, contributing 193 million USD to the state economy in 2019 [1]. They are grown extensively during the spring and summer seasons on open fields. Cantaloupe (*Cucumis melo* var. cantalupensis Naudin) and watermelon (*Citrullus lanatus*) are commercially produced and are concentrated in the southern part of the state. Other cucurbits produced in the state include cucumber (*Cucumis sativus* L.), honeydew (*Cucumis melo* L. (Inodorus Group) ‘Honey Dew’), muskmelon (*Cucumis melo*), pumpkin, yellow squash, and zucchini (*Cucurbita pepo* L.).

In recent years, cucurbit production in Georgia has incurred severe annual losses due to increased whitefly (*Bemisia tabaci* Gennadius) populations and the incidence of viruses transmitted by them. Three whitefly-transmitted viruses (WTVs), the cucurbit leaf crumple virus (CuLCrV) (Genus *Begomovirus,* family Geminiviridae) [2], the cucurbit yellow stunting disorder virus (CYSDV) [3], and the cucurbit chlorotic yellows virus (CCYV) (Genus *Crinivirus,* family Closteroviridae) [4] are reported on major cucurbits, including cantaloupe, cucumber, squash, watermelon, and zucchini in Georgia [3,5]. Recently, the presence of watermelon crinkle leaf-associated virus 1 (WCLaV-1) was reported in watermelons in Georgia [6]. Furthermore, whitefly-transmitted squash vein yellowing virus (SqVYV) [7] and watermelon crinkle leaf-associated virus 2 (WCLaV-2) [8,9] are a concern to watermelon production in the state due to their recent reports in the neighboring state of Florida.

Although much of the damage due to whitefly-transmitted viruses (WTVs) is inflicted on the fall-grown cucurbits, their incidence is assumed to build up slowly over spring and summer, with an increasing whitefly population on fall-grown cucurbits. Furthermore, the majority of the WTVs of cucurbits previously identified in Georgia, USA were on fall-grown vegetables [5]. The objectives of this study was: (1) to determine if spring-grown cantaloupe and watermelon are a reservoir of WTVs that cause damage in the fall and (2) to determine the diversity of viruses infecting spring-grown cucurbits and assess their prevalence in Georgia. Viruses present in the watermelon and cantaloupe samples were identified by high throughput sequencing (HTS) of small (s) RNAs which enable simultaneous screening and detection of known and novel viruses [5]. This technique employs the use of RNA interference (RNAi), a natural antivirus defense mechanism present in the host cell, to generate small RNAs that are 21–24 nt long for the analysis of the viral population present in a tested plant sample [10]. The technique has become an important tool and has been used widely in the diagnosis, identification, and characterization of viruses in recent years [5,11,12,13,14,15,16,17,18,19].

## 2. Materials and Methods

### 2.1. Survey Route and Sample Collection

**Figure 1:**
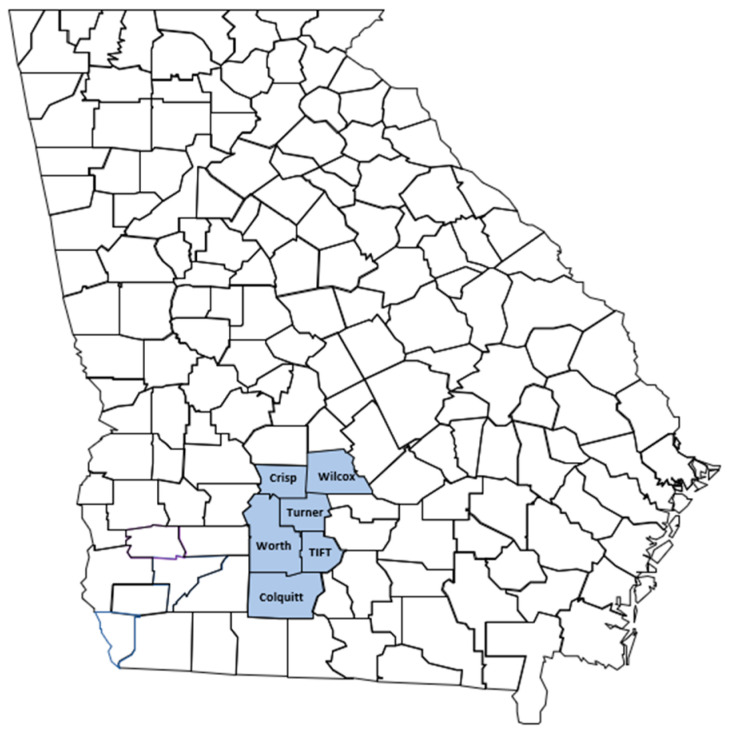
Map of Georgia showing counties from which melon samples were collected during spring 2021 survey.

In spring 2021, an extensive survey was conducted on open fields in Colquitt, Crisp, Tift, Turner, Wilcox, and Worth counties (Figure 1), which represent the major cantaloupe and watermelon-producing areas of the state. On symptomatic watermelons, 60 foliar tissues each were collected from both Crisp and Wilcox counties, 40 samples each were collected from both Turner and Tift counties, while 25 and 20 samples were collected from Colquitt and Worth counties, respectively. Forty symptomatic cantaloupe samples each were collected from both Turner and Tift counties. A total of 245 watermelons and 80 cantaloupes were collected to be processed by PCR and reverse transcription-polymerase chain reaction (RT-PCR). In addition, seven samples representing the symptoms observed on the field were also collected, surface sterilized three times with distilled water, and frozen on dry ice for HTS. Samples were transported on ice to the Crop Virology Laboratory, University of Georgia, Tifton Campus, and stored at −80 °C for further laboratory assays. In addition to this, 43 symptomatic leaf tissues of the watermelon collected in the fall of 2019 in Colquitt County were also tested for the presence of viruses detected by HTS.

### 2.2. RNA Extraction and HTS of Small RNA

**Figure 2:**
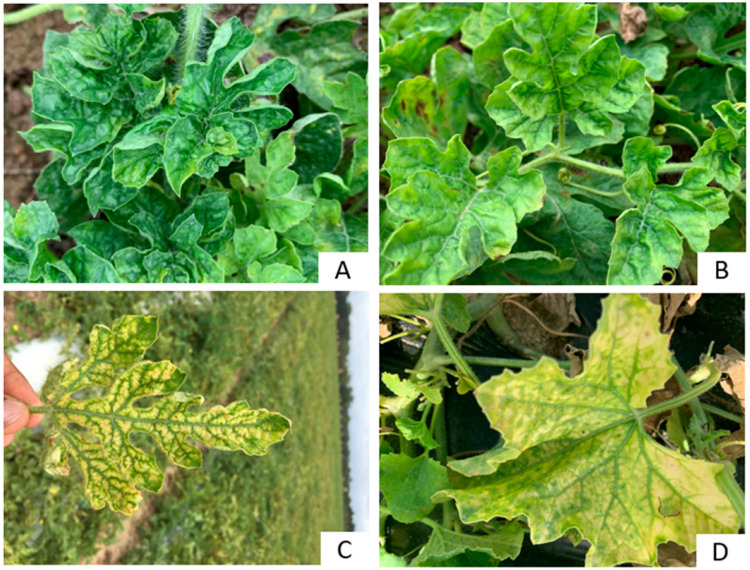
Symptoms observed on the field during the survey include: on watermelon (**A**–**C**), leaf crinkling and bunchy top with upward curling appearance of young leaves (**A**), crinkling of young leaves with mild yellowing (**B**), severe interveinal chlorosis of the leaf (**C**). On cantaloupe, yellowing and interveinal chlorosis on the older leaf (**D**). Watermelon (**A**,**B**) and cantaloupe (**D**) was collected in spring 2021 and watermelon (**C**) was collected in fall 2019.

Leaf tissues from one cantaloupe and six watermelon samples representing the distinct symptoms (Figure 2) observed on the field were macerated in liquid nitrogen. Total RNA was extracted using Trizol (Invitrogen, Carlsbad, CA, USA) following the manufacturer’s instructions. RNA integrity (RIN) was analyzed using a Qubit 4.0 Fluorometer (Thermo Fisher Scientific, Waltham, MA, USA) and samples with RIN values above 8.0 were sent to Beijing Genomic Institute (BGI, San Jose, CA, USA) on dry ice for HTS of sRNA. A DNA Nanoball (DNB) small RNA sequencing platform was used for the construction of small RNA libraries with a single-end read of 1 × 50 bp (BGI, Hong Kong, China).

### 2.3. Analysis of HTS Data

Analysis of small RNA to identify the virome community in the samples were performed with CLC Genomics Workbench 21 (Qiagen, Redwood City, CA, USA). Sequences with low-quality adapters and those having more than two ambiguous nucleotides were removed. Reads with 18–30 nucleotides in length were filtered and analyzed. de novo assembly of the contigs (minimum 50 nt) was carried out using published parameters (mapping mode-create simple contig sequences(fast), mismatch cost-2, insertion cost-3, deletion cost-3, alignment mode-local, minimum contig length-50) [19]. A local virus database was created from the National Center for Biotechnology Information (NCBI) (http://www.ncbi.nlm.nih.gov/genome/viruses) (downloaded on 6 January 2021) using the Create Database feature of CLC Genomics Workbench 21. Contigs were compared against all sequences in the database for possible similarities using BLASTn [20] with default settings in CLC Genomics Workbench 21. Contigs that mapped to non-plant virus sequences were not considered for further analysis.

### 2.4. Total Nucleic Acid Extraction and Virus Detection by PCR and RT-PCR

Total nucleic acid (TNA) isolation was done using 100 mg of symptomatic leaf tissue with magnetic bead mill technology which allowed simultaneous processing and preparation of a large number of samples for the detection of viruses. Samples were grinded using 4 M guanidine thiocyanate (GTC) buffer (pH 5.0) with Bead Mill 24 Homogenizer (Thermo Fisher Scientific, Waltham, MA, USA). TNA was isolated from homogenized samples with MagMAX 96 viral RNA kit using the KingFisher Flex Purification System (Thermo Fisher Scientific, Waltham, MA, USA) with the manufacturer’s instructions without the DNase treatment. More specific details of the RNA isolation protocol have been described earlier [5]. The quantity and purity of isolated TNA were assessed using a NanoDrop Spectrophotometer (Thermo Fisher Scientific, Waltham, MA, USA). They were stored at −80 °C for further laboratory analysis, pending the results from HTS.

Complementary DNA (cDNA) was synthesized using 1 µL of 10 µM random primers denatured with 10 µL TNA at 70 °C for 5 min. This was chilled at 4 °C. Subsequently, reverse transcription master mix was prepared using 8 µL of reverse transcriptase buffer, 2 µL of 100 mM DTT, 4 µL of 10 mM dNTPs mix, 2 µL Superscript III (200 U/µL) (Invitrogen, Carlsbad, CA, USA), 1 µL RNase out (50 U/µL), and 3 µL RNase free water with a final volume of 20 µL.

PCR reaction was done using 5 µL of 5X GoTaq green buffer with MgCl_2_, 0.5 µL dNTPs (10 mM), 0.5 µL each of forward and reverse primers (10 µM), 16.25 µL of RNase free water, 0.25 µL GoTaq polymerase (5 U/µL) (Promega, Madison, WI, USA), and 2 µL cDNA or TNA, amounting to a final volume of 25 µL. PCR was performed with initial denaturation at 95 °C for 2 min, followed by 35 cycles each of 95 °C for 30 s, annealing for 30 s, 72 °C for 1 min/kb, with the final extension of 72 °C for 5 min. All primers and annealing temperatures used for the detection of each virus are given in Table 1. cDNA preparations and PCR reactions were performed in a T100 thermal cycler (Bio-Rad, Hercules, CA, USA). Plasmids with the fragments of the virus being amplified by the primers used for testing were used as positive controls for CCYV, CYSDV, and CuLCrV. While the subset of samples used for HTS and in which CmAV1, CmCV, CmEV, and WCLaV-1 were detected were used as controls for these viruses. Leaf tissue of yellow squash plants grown in an insect-proof cage in the greenhouse were used as negative controls. Water was also used as a no template control. The final PCR products were analyzed on 1% agarose gel horizontal electrophoresis with 1X TAE buffer containing Gel Red (Biotium, Fremont, CA, USA). The PCR products were gel purified and Sanger sequenced for the confirmation of the target amplification. The sequences were annotated and submitted to NCBI GenBank.

### 2.5. Construction of Consensus Viral Genome Sequences

Reference-based mapping was used to assemble the consensus sequences using CLC Genomics Workbench 21. Small RNA reads were aligned with the reference sequences of viruses potentially present in the samples. Consensus sequences were assembled from the mappings with the following parameters: mismatch cost = 2, insertion cost = 3, and deletion cost = 3. For graphical visualization of coverage in each region of the assembled genome, read tracks showing maximum, minimum, and average coverage values were created. The alignment of consensus sequences with the reference genome was inspected for discrepancies with CLC Genomics Workbench 21.

## 3. Results

### 3.1. Symptoms

Symptoms resembling those caused by viruses were observed on watermelons in all fields of six counties surveyed in spring 2021. The main symptoms observed on spring-grown watermelons were thickened, wrinkled leaves with a severe bunchy top and upward curling, accompanied by a mild yellowing appearance (Figure 2A) in Wilcox County. Similar foliar symptoms were also observed on Tift County watermelon fields (Figure 2B) but with less crinkling and bunchy appearance of the leaves. Watermelon samples collected in the fall of 2019 from Colquitt County displayed symptoms of severe interveinal chlorosis, mild crumpling, and yellowing (Figure 2C).

Cantaloupe plants from the two counties surveyed in spring 2021 displayed yellow mottling with interveinal chlorosis (Figure 2D). These symptoms were observed in both Tift and Turner counties. Symptoms observed on cantaloupe were more restricted to the crown, while on watermelon, they were evenly distributed except for the bunchy and wrinkling appearance restricted to the young leaves.

### 3.2. Viruses Detected by HTS and Their Characteristics

sRNA libraries were constructed from one cantaloupe and six watermelon samples. After quality control and removal of adapters, a total of 17 to 28 million reads were retained (Table 2). All samples had a similar length distribution, with 21–24 nt sRNAs being the most abundant. Contigs with a minimum length of 75 bp were assembled and compared with virus sequences on the NCBI database for identification using BLASTn. Only viruses infecting plants were further analyzed.

**Figure 3:**
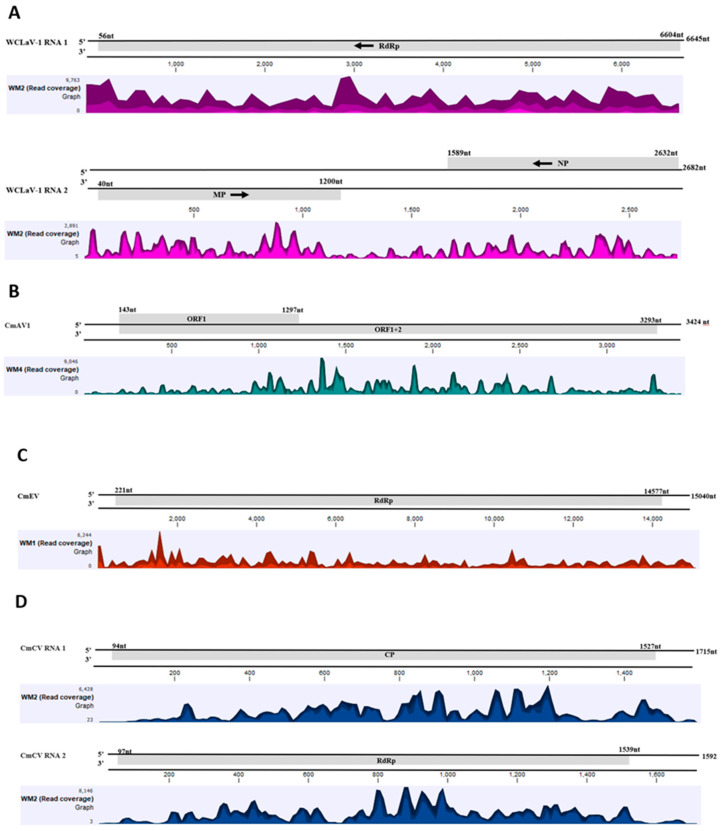
Read coverage maps of the virus genomes detected by HTS of small RNAs of symptomatic watermelon and cantaloupe from Georgia. Watermelon crinkle leaf-associated virus 1 (WCLaV-1) (**A**), cucumis melo amalgavirus 1 (CmAV1) (**B**), cucumis melo endornavirus (CmEV) (**C**), and cucumis melo cryptic virus (CmCV) (**D**). Genome positions of the virus are presented to scale above the histograms and the coverage in number of reads is represented on the *Y*-axis. Within the specified aggregation bucket, the colors mean: the maximum, average, and the minimum coverage values (read counts), from top to bottom.

Four different plant RNA viruses were identified after *de novo* assembly and BLASTn searches against the NCBI database. One of them was the recently reported watermelon crinkle leaf-associated virus 1 (WCLaV-1) [6] and the other three were persistent viruses: cucumis melo amalgavirus 1 (CmAV1), cucumis melo cryptic virus (CmCV), and cucumis melo endonavirus (CmEV). The large number of sRNA reads aligning to the genome (Table 2) and covering the entire length of their genome (Figure 3) added confidence to their identification. The percentage of viral sRNA sequences varied from sample to sample (Table 2). CmAV1 was detected in one cantaloupe and four out of six watermelon samples. CmCV and CmEV were detected only in watermelon samples, while WCLaV-1 was detected in one sample each of watermelon and cantaloupe.

In addition, reads from each sample were also aligned to the reference sequences of WTVs, CCYV, CuLCrV, and CYSDV (CuLCrV: DNA A-NC_002984, DNA B-NC_002985; CCYV: RNA1-NC_018173.1, RNA2-NC_018174.1; CYSDV: RNA1-NC_004809.1, RNA2- NC_004810.1), however these viruses were not detected in any samples collected in the spring, either on watermelon or cantaloupe.

Near complete genomes of CmAV1, CmCV, CmEV, and WCLaV-1 were assembled from the sRNA sequences. The nucleotide sequence identity of CmAV1 isolates from watermelon (OM751927) ranged from 99.18 to 99.85% to that of CmAV1 reported in China (MH479774). The genome of CmCV from watermelon in Georgia was 99.69% identical with RNA1 (MH479772) and 99.94% identical with RNA 2 (MH479773) with the isolate from China. CmEV sequences on watermelon matched 96.68% with that of CmEV reported from Mississippi (KT727022). The watermelon (RNA1-OM751928 and RNA2-OM751930) and cantaloupe isolate of WCLaV-1 Georgia shared 99.71% and 99.73% identity with RNA 1 and 99.30% with RNA 2 of WCLaV-1 isolates reported in Brazil in BLASTn analysis (Table 2).

### 3.3. Prevalence and Distribution of the Viruses

Foliar tissues of watermelon collected in the spring of 2021 (*n* = 245) and the fall of 2019 (*n* = 43) and cantaloupe collected in spring 2021 (*n* = 80) were tested for the presence of viruses detected by HTS in this study (Table 2), as well as the WTVs previously reported to infect cucurbits in the region (Table 3). The presence of viruses, detected via HTS, were validated using RT-PCR on the portion of seven symptomatic samples sent for HTS.

In the samples collected in spring 2021, CmAV1 was detected by RT-PCR on both watermelon and cantaloupe samples in different counties. CmAV1 was detected in a high percentage of watermelon samples in Crisp (78%), Tift (50%), Turner (68%), and Wilcox (62%) counties, with only 5% from Worth County. On cantaloupe, CmAV1 was detected in 15% and 3% of samples from Tift and Turner counties, respectively. CmEV was detected in 100% of the cantaloupe samples from Tift and Turner counties and 20% of the total watermelon samples tested from Turner County. WCLaV-1 was also detected on watermelons in all counties surveyed, except for Crisp County. The percentage of samples in which WCLaV-1 was detected varied in each county. It was detected in 8% of the total samples tested from Colquitt, 5% of samples from Worth, 28% of samples from Wilcox, 83% of samples from Turner and 98% of samples from Tift County (Table 3). The presence of WCLaV-1 was only detected on watermelon and not in cantaloupe using RT-PCR, even though WCLaV-1 was detected from a cantaloupe sample by HTS. However, CmCV was not detected from any samples tested using RT-PCR. Mixed infection of more than one virus was also identified (Supplementary Material Table S1). Surprisingly, none of the WTVs, including CCYV, CYSDV, CuLCrV, previously identified in the cucurbits in Georgia, were detected in any of the samples tested.

RT-PCR and PCR analysis of the symptomatic foliar tissues of the watermelon collected in the fall of 2019 revealed the presence of CCYV in 79%, CYSDV in 2%, and CuLCrV in 81% of the total samples tested (Table 3). Dual infection of CCYV and CuLCrV was detected in 67% of the total samples, while the mixed infection of the three viruses was found in 2% of the total samples tested (Supplementary Material Table S1). However, viruses detected in spring 2021, CmAV1, CmCV, CmEV, and WCLaV-1, were not detected in any of the samples collected in the fall of 2019. The partial sequence of the CCYV coat protein (MW915456; MW915457) and heat shock protein (MW915459; MW915460), the CmAV1 RNA-dependent RNA polymerase (ON364007), and the CmEV polyprotein (ON364008) from this study were submitted to the NCBI GenBank.

## 4. Discussion

The application of HTS has become an important tool for detecting and identifying known and novel viruses in plant samples. In addition, many viruses infecting important food crops have been characterized [21,22,23,24,25,26,27,28,29]. Unlike traditional assays like hybridization, ELISA, and PCR, metagenomic diagnostic techniques are unbiased and can detect pathogens without prior knowledge of their existence [27,28,29]. In many cases, the virome of a host plant has been used as a basis for field diagnosis [5,26]. siRNA sequencing has the advantage of detecting all types of viral and viroid genomes [26,29]. In this study, surveys were conducted in the major cantaloupe and watermelon growing counties in Georgia and HTS was employed to explore the diversity of viruses infecting cantaloupe and watermelon. A large number of samples collected during the survey were tested by RT-PCR to determine the distribution and prevalence of viruses identified by HTS.

sRNA sequences from one cantaloupe and six watermelon samples were generated by HTS. Four different RNA viruses (CmAV1, CmCV, CmEV, and WCLaV-1) were identified from the sRNA sequences. Among the four viruses identified, only WCLaV-1 is reported to cause symptoms on watermelon, while CmAV1, CmCV, and CmEV express asymptomatic infections. WCLaV-1 was detected in one of the six watermelon samples analyzed by HTS. This virus was recently identified in Georgia on watermelon [6] and the samples from which it was detected showed symptoms of mosaic, crinkling, and bunching (Figure 2A), as described previously [8,30,31]. Based on the molecular features and phylogenetic reconstructions, WCLaV-1 has been provisionally assigned to the genus Coguvirus (family *Phenuiviridae*) [30]. Since its discovery in China [30] in 2017, the virus has been found in Brazil [31] and the USA [8]. The isolate of WCLaV-1 from Georgia (RNA1-OM751928 and RNA2-OM751930) was identical to those from Brazil (100% for RNA 1-LC636070 and 99% for RNA 2-LC636069;) and China (99% for RNA 2-MW751424.1) based on the nucleotide sequence of the near-complete genome sequence of RNA 1 and RNA 2. Even though WCLaV-1 was identified in the USA only recently, the virus appears to be widely distributed in the state and was detected from most of the counties surveyed in spring 2021.

WCLaV-1 has been found to be consistently associated with WCLaV-2 wherever it was detected, including Brazil [31], China [30], and the USA [8,9]. However, in this study, WCLaV-2 was not detected in any of the samples tested either by HTS or RT-PCR. WCLaV-1 and WCLaV-2 are rather recently discovered viruses and much has yet to be learnt about both, including vector relations.

CmAV1, CmCV, and CmEV are classified as persistent viruses based on their lifestyle. They infect economically important crops, although they do not cause any obvious symptoms and remain in the host for a long time [32]. So far, most members of this plant virus group identified have a double-stranded RNA genome. They generally do not code for movement proteins or move from cell to cell. These viruses are almost 100% vertically transmitted by seed in hosts and not horizontally by insects or grafting.

Plant persistent viruses are not well-studied. Their genomes code for RNA-dependent RNA polymerase (RdRp) and a coat protein. Endornaviruses are an exception since they do not have domains that code for a coat protein and instead, only code for a polyprotein which functions as RdRp [13]. Cryptic viruses, which are currently classified in the family *Partitiviridae* [33], were the earliest members identified in this group in the 1960s and 1970s [34]. Other viruses with similar characteristics were discovered and described later [35,36,37,38]. Earlier methods of the discovery of persistent viruses from dsRNA preparations were not efficient. Particularly in cases like endornaviruses, in which the members do not form classic virions (“capsid-less viruses”) [23,32], their naked RNA can go unnoticed while contaminating DNA in dsRNA analysis [32]. Metagenomic studies have accelerated the discovery of new viruses in this category [13,37,38,39,40,41,42,43,44,45,46]. In addition, the genomes and sequences generated from these studies will improve our understanding of these viruses, including phylogeny, host relation, and transmission.

CmEV belongs to the genus *Endornavirus* and family *Endornavirudae* [22], and CmAV1 belongs to the genus *Amalgaviruses* and family *Amalgaviridae* [21]. Endornaviruses are persistent within the host and infect every tissue of the plant [34,35,36]. Pepper endornaviruses were found in all cultivars of bell pepper and could be transmitted to other cultivars by crosses [47]. In contrast, CmEV was identified in plants belonging to three different genera in the family Cucurbitaceae (*Cucumis*, *Luffa* and *Praecitrullus*), which are not cross-compatible [22]. In this study, CmEV was identified from watermelon (*C. lanatus*), which is not cross-compatible with either of the three genera from which CmEV was reported earlier. These findings suggest the long association of endornaviruses with their hosts and their co-divergence with hosts since they are not transmitted horizontally. It is also the first time CmEV and CmAV1 have been identified on watermelon (Table 3).

All virus isolates identified in this study had very high genetic similarity with isolates of corresponding viruses described earlier. The phylogenetic relations and detailed genome characteristics of CmAV1 [21], CmCV [48,49], CmEV [22], and WCLaV-1 [30] have been described earlier and are not repeated here.

Although sequences of CmCV and WCLaV-1 were detected on watermelon and cantaloupe in HTS (Table 2), their presence could not be verified in any of the samples by RT-PCR. This could have resulted from cross-contamination during RNA preparation, which is not uncommon in HTS [50,51,52,53]. This suggests the importance of not relying on HTS alone and the reliability of the use of alternate methods to confirm presence of a virus identified by HTS. Similarly, no virus was detected in some samples that displayed virus-like symptoms. This could be due to other factors, including environmental conditions, nutrient imbalances, chemicals, or non-viral pathogen infections.

Overall, this study did not find any evidence of a buildup of previously reported WTVs viz CuLCrV, CYSDV, and CCYV affecting cucurbits on spring-grown cantaloupe and watermelon in Georgia. This indicates non-crop hosts are likely sources of inoculum for the buildup of WTVs in the fall crops. After the fall season harvest of cucurbits, the whiteflies migrate to winter brassica crops and weeds, likely carrying the viruses with them [54]. However, the spring population of whiteflies is much lower than what is seen in the fall [55] and may not be enough to spread the viruses from alternate crop or weed hosts, such as the wild radish [56], to infect the spring cucurbits.

Nevertheless, spring cantaloupe and watermelon in Georgia support a diverse array of viruses, including the recently discovered WCLaV-1 and three persistent viruses. WCLaV-1 appears to have spread in Georgia since it was detected on watermelon in five different counties. Being a rather recently discovered virus, biological properties, including vector relations, are unknown. Watermelon is an economically important crop in Georgia and worldwide. Further studies are required to better understand WCLaV-1 and its impact on watermelon production. The mode of spread/transmission, including vectors, if any, crops and weed hosts of the virus, and most importantly, the economic impact of the virus on its crop hosts are important questions to be answered.

## 5. Conclusions

Applications of HTS and surveillance reveal the presence of new persistent plant viruses in cantaloupe and watermelon in GA. CmAV1 and CmEV are widespread and prevalent in the region, along with the newly identified WCLaV-1 in GA. The WTVs (CCYV, CYSDV, CuLCrV, and SqVYV) were not detected in the spring cantaloupe and watermelon, suggesting these viruses may overwinter in another crop and non-crop host. These results present the first report of CCYV, CmAV1, and CmEV on watermelon in Georgia, USA. The impact of these viruses on the agroecosystem in the state is yet to be investigated.

## Figures and Tables

**Table 1:** Primers used for PCR and RT-PCR for confirmation of the viruses.

Primer Name	Sequences 5′–3′	Tm (°C)	Amplicon Size	References
CmAV1-2459F	AACCTCCCACATTCTGGA	55	740	[21]
CmAV1-3189R	TCCAGTCAGCATAGGTCTCC			
CmEV-1F	ACCCCACTATTAGATATGCTAAGGTC	55	680	[22]
CmEV-1R	CTCCAGGAGTAAGATATAATGTAACCG			
CmCV-109F	ACTGAAGGATGAGTTCGCA	55	640	[21]
CmCV-751R	CCATCGGCATTCAGAACT			
CCYV_RDRP_1515	CTCCGAGTAGATCATCCCAAATC	62	953	[4]
CCYV_RDRP_1515	TCACCAGAAACTCCACAATCTC			
CuLCrV CP 259 F	TCAAAGGTTTCCCGCTCTGC	58	588	[5]
CuLCrV CP 846 R	TCCTGCTTCCTGGTGGTTGTAG			
CYSDV_RDRP_1542	TTTCGGCTCCCAGAGTTAATG	58	492	[23]
CYSDV_RDRP_1542	CGATCTCCGTGGTGTGATAAG			
WCLaV-1F	GGTGAGTTAGTGTGTCTGAAGG	55	881	[8]
WCLaV-1R	GAGGTTGCCTGAGGTGATAAG			

Abbreviations used for viruses: Watermelon crinkle leaf-associated virus 1 (WCLaV-1), cucumis melo amalgavirus (CmAV1), cucumis melo endornavirus (CmEV), cucumis melo cryptic virus (CmCV), cucurbit chlorotic yellows virus (CCYV), cucurbit yellow stunting disorder virus (CYSDV) and cucurbit leaf crumple virus (CuLCrV).

**Table 2:** Viruses identified in sRNA reads from watermelon and cantaloupe samples collected in spring from Georgia and their characteristics.

Virus Detected	Sample ID & Location	Genome Size of Refseq (nt)	Total sRNA Reads	Reads Matching to Virus	Coverage (%)	Nucleotide Identity (%)
CmAV1	WM3	3424	17,110,156	54,623 (0.31)	3395 (99.1)	99.18
	Wilcox					
	WM4	3424	19,040,070	189,757 (0.99)	3397 (99.2)	99.24
	Wilcox					
	CA	3424	18,645,801	27,819 (0.14)	3419 (99.8)	99.85
	Turner					
	WM2	3424	28,380,401	16,477 (0.05)	3398 (99.2)	99.24
	Wilcox					
	WM5	3424	21,552,412	30,070 (0.13)	3398 (99.2)	99.27
	Wilcox					
CmCV	WM2	1592 RNA 1 1715 RNA 2	28,380,401	144,683 (0.5)	1587 RNA 1 (99.9)	99.94 RNA 1
	Wilcox				1714 RNA 2 (99.6)	99.60 RNA 2
CmEV	WM 1	15078	18,645,801	273,320 (1.46)	14,577 (96.6)	96.68
	Tift					
WCLaV-1	WM2	6645 RNA 1 2682 RNA 2	28,380,401	557,592 (1.96)	6599 RNA 1 (99.3)	99.71(RNA 1)
	Wilcox				2678 RNA 2 (99.8)	99.30 (RNA 2)
	CA	6645 RNA 1 2682 RNA 2	18,645,801	239,859 (1.28)	6601 RNA 1 (99.3)	99.73 (RNA 1)
	Turner				2678 RNA 2 (99.8)	99.30 (RNA 2)

All samples except WM3 and WM4 were leaves. WM3 was fruit skin and WM4 was peduncle from watermelon. Viruses detected: watermelon crinkle leaf-associated virus 1 (WCLaV-1), cucumis melo amalgavirus (CmAV1), cucumis melo endornavirus (CmEV), cucumis melo cryptic virus (CmCV). Complete genome: Genome length of virus sequence with the highest identity in BLAST. CmEV (KT727022, Mississippi); WCLaV-1 (RNA 1, LC636068.1; RNA 2 LC636069, Brazil); CmAV1 (MH479774, China); CmCV (MH479773, China). Reads matching the virus: values in parenthes is represent the percentage of total reads aligning with the virus genome. Coverage: percentage of the GenBank genome covered by contigs assembled from the sample. Percentage nucleotide identity: identity to the GenBank sequence with the highest nucleotide identity in BLAST of all contigs aligned to the sequence.

**Table 3:** Incidence and prevalence of viruses from fall 2019 and spring 2021 cantaloupe and watermelon samples in Georgia as detected by PCR and RT-PCR.

Virus	2021								2019	Virus Detected in Number of Samples
	Watermelon						Cantaloupe		Watermelon	
	Colquitt	Crisp	Worth	Wilcox	Turner	Tift	Turner	Tift	Colquitt	
WCLaV-1	2(8)	-	1(5)	17(28)	33(83)	**39 (98)**	-	-	-	92
CmAV1	-	**47 (78)**	1(5)	37(62)	27(68)	20(50)	1(3)	6(15)	-	139
CmEV		-	-	-	8(20)	-	**40 (100)**	**40 (100)**	-	88
CCYV	-	-	-	-	-	-	-	-	**34 (79)**	34
CYSDV	-	-	-	-	-	-	-	-	**1 (2)**	1
CuLCrV	-	-	-	-	-	-	-	-	**35 (81)**	35
CmCV	-	-	-	-	-	-	-	-	-	-
Total sample tested	25	60	20	60	40	40	40	40	43	

Virus acronyms used: Watermelon crinkle leaf-associated virus 1 (WCLaV-1), cucumis melo amalgavirus (CmAV1), cucumis melo endornavirus (CmEV), cucurbit chlorotic yellows virus (CCYV), cucurbit yellow stunting disorder virus (CYSDV), and cucurbit leaf crumple virus (CuLCrV). All samples collected in this study were also tested for squash vein yellowing virus (SqVYV), watermelon crinkle leaf-associated virus 2 (WCLaV-2), but none were detected. The number of samples in which a virus was detected and their percentages (in parenthesis) are presented. The numbers for the virus detected at the highest frequency on a crop in a particular year are shown in bold.

## Data Availability

Not applicable.

## Supplementary Materials

The following supporting information can be downloaded at: https://www.mdpi.com/article/10.3390/v14061310/s1, Table S1: Prevalence and distribution of mixed infection of the viruses detected during 2019 and 2021 cantaloupe and watermelon survey.
