# Supplementary material for: Persistent, and Asymptomatic Viral Infections and Whitefly-Transmitted Viruses Impacting Cantaloupe and Watermelon in Georgia, USA
Source: Viruses. 2022 Jun 15;14(6):1310. doi: 10.3390/v14061310 (PMC9227350; doi:10.3390/v14061310)
Supplement: Supplementary file 1 [file viruses-14-01310-s001.zip › viruses-1735795-supplementary.pdf]

Supplementary Material Table S1: Prevalence and distribution of mixed infection of the v*iruses* detected during 2019 and 2021 cantaloupe and watermelon survey

Virus	2021								2019
	Watermelon						Cantaloupe		Watermelon
	Colquitt	Crisp	Worth	Wilcox	Turner	Tift	Turner	Tift	Colquitt
CCYV + CuLCrV	-	-	-	-	-	-	-	-	29(67)
CmAV + CmEV	-	-	-	-	-	-	3(1)	15(6)	-
CmAV + WCLaV-1	-	-	-	-	50(20)	50(20)	-	-	-
CmEV + WCLaV-1	-	-	-	-	10(4)	-	-	-	-
CuLCrV + CCYV + CYSDV	-	-	-	-	-	-		-	1(2)
CmAV + CmEV + WCLaV-1	-	-	-	-	10(4)	-	-	-	-
**Total number of samples tested**	25	60	20	60	40	40	40	40	43

Virus acronyms used: cucurbit chlorotic yellows virus (CCYV), cucurbit leaf crumple virus (CuLCrV), cucumis melo amalgavirus (CmAV), cucumis melo endornavirus (CmEV). cucurbit yellow stunting disorder virus (CYSDV) and watermelon crinkle leaf-associated virus 1 (WCLaV-1).
